# Clinical reasoning education in the clerkship years: A cross-disciplinary national needs assessment

**DOI:** 10.1371/journal.pone.0273250

**Published:** 2022-08-18

**Authors:** Jonathan G. Gold, Christopher L. Knight, Jennifer G. Christner, Christopher E. Mooney, David E. Manthey, Valerie J. Lang

**Affiliations:** 1 Department of Pediatrics and Human Development, Michigan State University College of Human Medicine, East Lansing, Michigan, United States of America; 2 Department of Internal Medicine, University of Washington School of Medicine, Seattle, Washington, United States of America; 3 Baylor School of Medicine, Houston, Texas, United Sates of America; 4 Department of Internal Medicine, University of Rochester School of Medicine, Rochester, New York, United States of America; 5 Department of Emergency Medicine, Wake Forest School of Medicine, Winston-Salem, North Carolina, United States of America; University of Texas Southwestern Medical Center at Dallas, UNITED STATES

## Abstract

**Background:**

Improving clinical reasoning education has been identified as an important strategy to reduce diagnostic error—an important cause of adverse patient outcomes. Clinical reasoning is fundamental to each specialty, yet the extent to which explicit instruction in clinical reasoning occurs across specialties in the clerkship years remains unclear.

**Method:**

The Alliance for Clinical Education (ACE) Clinical Reasoning Workgroup and the Directors of Clinical Skills Courses (DOCS) Clinical Reasoning Workgroup collaborated to develop a clinical reasoning needs assessment survey. The survey questionnaire covered seven common clinical reasoning topics including illness scripts, semantic qualifiers, cognitive biases and dual process theory. Questionnaires were delivered electronically through ACE member organizations, which are primarily composed of clerkship leaders across multiple specialties. Data was collected between March of 2019 and May of 2020.

**Results:**

Questionnaires were completed by 305 respondents across the six organizations. For each of the seven clinical reasoning topics, the majority of clerkship leaders (range 77.4% to 96.8%) rated them as either moderately important or extremely important to cover during the clerkship curriculum. Despite this perceived importance, these topics were not consistently covered in respondents’ clerkships (range 29.4% to 76.4%) and sometimes not covered anywhere in the clinical curriculum (range 5.1% to 22.9%).

**Conclusions:**

Clerkship educators across a range of clinical specialties view clinical reasoning instruction as important, however little curricular time is allocated to formally teach the various strategies. Faculty development and restructuring of curricular time may help address this potential gap.

## Introduction

Diagnostic error is an underrecognized cause of adverse patient outcomes, affecting 1 in 20 adults in outpatient settings per year and contributing to approximately 10% of patient deaths in the United States [1, 2]. Effective clinical reasoning is essential to providing accurate and timely diagnoses and treatments in all clinical specialties, yet little is known about what aspects of clinical reasoning are taught across clerkship specialties in undergraduate medical education. Understanding curricular gaps is a foundational step in designing integrated curriculum to prepare medical students to be more effective diagnosticians.

Clinical reasoning may be defined as “the cognitive and non-cognitive process by which a healthcare professional consciously and unconsciously interacts with the patient and the environment to collect and interpret patient data, weigh the benefits and risks of actions, and understand patient preferences to determine a working diagnostic and therapeutic management plan whose purpose is to improve a patient’s well-being” [3, 4]. There are multiple underlying theories which explain clinical reasoning, and dual process theory [5], embodied cognition [6], situated cognition [7, 8], and distributed cognition [9] have emerged as informative and encompassing theories [10]. Dual process theory explains reasoning as occurring in a fast, heuristically-driven, intuitive manner (“Type 1”) or a slower, analytical manner (“Type 2”) [3]. Physicians tend to use fast reasoning when problems seem familiar to them, and slow reasoning for problems that appear to be out of their domain of expertise. An example of type 2 reasoning is Bayesian reasoning, in which the physician establishes a pretest probability for a diagnosis, then uses likelihood ratios to calculate a posttest probability. Embodied cognition highlights that clinical reasoning is not simply data processing, but occurs in human beings whose reasoning is shaped by their sensations and the impact of their own interactions with their environment [6]. Situated cognition theory places these clinical reasoning processes in context, and emphasizes that the physical environment (e.g. interruptions, time-pressure), patient factors (e.g. ability to communicate, animosity), and physician factors (e.g. fatigue, emotions, biases) can collectively influence the clinical reasoning process [7, 8]. Distributed cognition theory emphasizes how clinical reasoning often occurs across multiple individuals, teams, and systems [9].

Improving medical education pedagogy to purposefully teach clinical reasoning has emerged as a path to reducing diagnostic error. In 2015 the National Academy of Medicine (NAM) published the report “Improving Diagnosis in Healthcare” [1]. The report specifically identified the lack of clinical reasoning instruction in medical education as a contributing factor to diagnostic error. One of the NAM’s core recommendations was to “enhance health care professional education and training in the diagnostic process”.

Although elements of clinical reasoning are mandatory in medical school curricula [11], it is unknown to what extent elements of clinical reasoning are deliberately planned across discipline-specific clerkships. Some facets of the diagnostic process—namely history taking, physical examination, and rudimentary differential diagnosis—are standard components of the pre-clerkship curriculum. In recent years, directors of clinical skills courses have begun to incorporate other aspects of clinical reasoning into their pre-clerkship courses [12]. One study of internal medicine clerkships suggested that clinical reasoning education is more ‘caught’ (i.e. learned through experience and role modeling) than ‘taught’ (i.e. part of a structured curriculum), finding that more than half of institutions had no formal sessions dedicated to clinical reasoning [13]. However, this study was conducted in only one specialty, and discipline-specific nuances in clinical reasoning preclude generalizations across disciplines [14]. Understanding more about clinical reasoning education across the breadth of clerkship experiences is an essential step in improving the process.

The purpose of this needs assessment was to provide a broader view of the current state of clinical reasoning education across clerkship specialties, extending beyond internal medicine to include other commonly required clerkships.

## Materials and methods

### Study design, setting and participants

We conducted a cross-sectional needs assessment survey of members of clerkship organizations that comprise the Alliance for Clinical Education (ACE): the Association of Directors of Medical Student Education in Psychiatry (ADMSEP), the Association of Professors of Gynecology and Obstetrics (APGO), the Clerkship Directors in Emergency Medicine (CDEM), the Clerkship Directors in Internal Medicine (CDIM), the Council on Medical Student Education in Pediatrics (COMSEP), the Consortium of Neurology Clerkship Directors (CNCD) and the Society of Teachers of Family Medicine (STFM). Members of these organizations include clerkship leaders and faculty affiliated with medical education at the clerkship level. Members of the Alliance for Surgical Education (ASE) were also invited but declined to participate.

### Survey development

A group of clerkship directors and faculty interested in clinical reasoning education in the clerkship years was recruited through the Alliance for Clinical Education (ACE). The group partnered with members of the Directors of Clinical Skills Courses (DOCS) organization. The overall goal of the collaboration was to study the teaching of clinical reasoning across the span of undergraduate medical education.

The combined group developed parallel surveys designed to address the goals of our combined group. The initial survey topics were drawn from a 2015 survey of internal medicine clerkship directors [13] and modified based on an updated literature search and our study goals to include items about both clerkship and pre-clerkship teaching. The seven clinical reasoning topics were chosen both to allow direct comparison with the earlier single-specialty survey and because they represent the most common concepts included in most studies of clinical reasoning education. We revised the items in an iterative fashion in a process aligned with that described by Artino et al. [15]. Workgroup members, as target participants for the survey, served as an initial focus group. The group worked synchronously and asynchronously on item selection and word choice until consensus was reached.

The survey was then piloted with members of the research team and the ACE Research Committee. Feedback was obtained from experts in clinical reasoning education and educators with expertise in survey design. The final survey included items assessing the perceived importance of including a variety of clinical reasoning topics within clerkship curricula, the degree to which these topics were included, barriers to inclusion, and the importance of including these topics in the pre-clerkship curriculum. (S1 Appendix) To ensure participant understanding of clinical reasoning concepts, we included definitions and examples of clinical reasoning topics (Table 1).

**Table 1:** Clinical reasoning concepts included in the survey.

Clinical Reasoning Concept	Definition	Example(s)
Semantic qualifier	Abstract, often binary terms that help narrow or specify the meaning of a symptom, sign, pathologic process or disease	Acute vs. chronic, moderate vs. severe, or unilateral vs. bilateral
Problem representation	Clinician’s synthesis of key discriminating aspects of the history, exam, and data; often expressed as a summary statement	60 year-old male man with history of type 2 diabetes and hypertension with acute onset of left-sided chest pain and diaphoresis
Illness scripts	Clinician’s mental representations of clinical findings, underlying pathology, diagnostic and treatment approaches, and prognosis associated with diseases	The representative findings for croup are: age 18 months to 3 years-old with barky cough, and stridor, and presentation from October to March
Dual processing theory	Description of cognitive processes as the interplay of non-analytic and analytical reasoning approaches, described as System 1 (fast, pattern recognition, experience-based) vs. System 2 (slow, deliberate, rational)	An expert sees a swollen calf in a post-op patient and diagnoses a DVT (system 1). A novice learner looks at a swollen leg and needs to consider a variety of causes, associated with pathophysiology to consider DVT as a diagnosis (system 2).
Heuristics	Mental shortcuts or rules of thumb used subconsciously in approaching a problem	A physician immediately thinks of influenza in a patient presenting with fever during flu season
Bayesian reasoning	Calculating the post-test probability using the pre-test probability and the likelihood ratio.	With a pretest probability of 25% for pulmonary embolus and a negative D-dimer test with a LR of 0.1, the post-test probability of PE is 3%
Cognitive bias	Dispositions or preferences that can affect judgments and decisions in a subconscious manner	Narrowly focusing on a single feature (sore throat) to support the diagnostic hypothesis (e.g. Strep pharyngitis), even if other or new information refutes it (cough, lack of exudate, lack of fever)

### Survey administration

The survey was administered to clerkship directors through their professional organizations. Several methods were used. Two organizations (COMSEP, ADMSEP) incorporated the survey items directly into their annual surveys. One organization (CDIM) created a special mid-cycle survey specifically for this purpose. Three organizations (STFM, CDEM, CNCD) shared a link to a Qualtrics survey with their membership and invited participation. For all groups, emails were sent to nonresponders.

The study was conducted between March of 2019 and May of 2020.

### Statistical analyses

We calculated descriptive statistics across study variables. To minimize potential for bias introduced by respondents who may be less familiar with their institution’s clerkship curriculum, we examined ratings across those who were clerkship leaders and other affiliated clerkship faculty. We examined differences in importance and inclusion of the seven clinical reasoning strategies across respondents designated as clerkship leaders (clerkship director, co-clerkship director, associate clerkship director, or site director) and those not in leadership roles using chi-square analyses. The chi-square threshold for statistical significance was set at p<0.01. All analyses were performed using Stata (16.1, StataCorp LLC, College Station, TX).

To provide a comparison between importance and extent to which clinical reasoning concepts are covered in respondents’ clerkships, we estimated a gap score as described by Cayea et al. [16]. For each clinical reasoning concept, we calculated the absolute difference between the percentage of respondents indicating that a concept was “covered in the clerkship” and the percentage indicating the concept was “extremely important” or “moderately important”. Thus, higher scores are indicative of a greater discordance between ratings of importance and inclusion in clerkships.

### Ethics

Each participant consented to participate in the study electronically prior to completing the survey. This study was determined exempt by the Michigan State University Human Research Protection Program as well as the Institutional Review Boards for CDIM, ADMSEP and COMSEP.

## Results

The overall response rate was 19% (305/1859) and ranged from 3% to 30% across specialties. The majority of survey respondents represented pediatrics (43.6%), internal medicine (33.1%) and family medicine (15.1%), followed by obstetrics and gynecology (3.6%), emergency medicine (3.0%) and neurology (1.6%). An additional 77 surveys were answered by representatives from psychiatry. However, the survey had been altered prior to administration, with the addition of an extra answer option on the items about importance of clinical reasoning concepts, and modification of the anchors on the items about whether the concepts were taught in the clerkship. The psychiatry data was therefore not included in the analyses, but responses to the relevant items are included in S2 Appendix. Approximately 77% of respondents identified themselves as clerkship leaders; the remainder had other roles affiliated with the clerkship. The majority of clerkships were over 6 weeks in length (Table 2).

**Table 2:** Respondent demographics.

Variable	n (%)
Medical specialty (n = 305)	
Neurology	5 (1.6)
Family medicine	46 (15.1)
Emergency medicine	9 (3.0)
Obstetrics/Gynecology	11 (3.6)
Pediatrics	133 (43.6)
Internal medicine	101 (33.1)
Role (n = 285)	
Clerkship leaders	220 (77.2)
Affiliated clerkship faculty	65 (22.8)
Clerkship length (n = 271)	
Less than 6 weeks	54 (19.3)
6 to 8 weeks	178 (65.7)
More than 8 weeks	24 (8.9)
Other	15 (5.5)

There were no statistically significant differences in ratings of importance of the various clinical reasoning strategies between clerkship leaders and non-leaders. However, clerkship leaders reported being more familiar with the inclusion of most clinical reasoning strategies in clerkship curriculum than non-clerkship leaders, with the exception of cognitive bias (p = 0.06). To ensure the analyses accurately reflected clerkship content, we elected to report results for the clerkship leaders only. The ratings of importance and inclusion provided by non-leaders are included in S2 Appendix.

Over 90% of respondents were familiar with all of the clinical reasoning concepts in the survey (Table 3). Concepts most frequently identified as extremely important were problem representations (74.1%), illness scripts (66.4%), cognitive bias (60.1%) and semantic qualifiers (49.1%). Respondents were least familiar with or more uncertain about dual processing theory (8.2%) and Bayesian reasoning (8.2%). Over 90% of respondents rated all clinical reasoning concepts as moderately or extremely important, with the exception of use and limitation of heuristics (86.6%), dual processing theory (77.4%) and Bayesian reasoning (80.4%). More respondents reported that semantic qualifiers, problem representations, and illness scripts were covered in the clerkship (range 62.0%-76.4%) than dual processing theory, Bayesian reasoning, and use/limitations of heuristics (range 29.4%-39.4%). While these latter three topics were considered moderately or extremely important by the majority of respondents, they accounted for the largest gaps between importance and coverage in the clerkships.

**Table 3:** Importance of teaching clinical reasoning concepts during the clerkship and degree to which they are included in clerkship phases (clerkship leaders)^a^.

Questionnaire item	Importance during clerkship (n = 220)				Included in clerkship (n = 217)				Gap^b^
	Not sure or unfamiliar n (%)	Not at all important n (%)	Moderately important n (%)	Extremely important n (%)	Not sure or unfamiliar n (%)	Not covered n (%)	Covered elsewhere n (%)	Covered in clerkship n (%)	(%)
Dual-processing theory	18 (8.2)	32 (14.6)	99 (45.0)	71 (32.4)	59 (27.1)	50 (22.9)	45 (20.6)	64 (29.4)	48.0
Bayesian reasoning	18 (8.2)	25 (11.4)	105 (48.0)	71 (32.4)	43 (19.9)	34 (15.7)	68 (31.5)	71 (32.9)	47.5
Use and limitations of heuristics	8 (3.7)	21 (9.7)	105 (48.4)	83 (38.2)	48 (22.2)	34 (15.7)	49 (22.7)	85 (39.4)	47.2
Cognitive bias	4 (1.8)	8 (3.7)	75 (34.0)	131 (60.1)	39 (18.1)	25 (11.6)	39 (18.1)	113 (52.3)	41.8
Illness scripts	2 (0.9)	6 (2.7)	66 (30.0)	146 (66.4)	19 (8.8)	19 (8.8)	40 (18.5)	138 (63.9)	32.5
Semantic qualifiers	9 (4.1)	5 (2.3)	98 (44.6)	108 (49.1)	24 (11.1)	25 (11.6)	33 (15.2)	134 (62.0)	31.7
Problem representations	4 (1.8)	3 (1.4)	50 (22.7)	163 (74.1)	19 (8.8)	11 (5.1)	22 (10.1)	165 (76.4)	20.4

^a^.Clerkship leaders include clerkship directors, co-clerkship directors, associate clerkship directors, and site directors

^b^Gap refers to the difference between the percentage of respondents indicating that a concept was “covered in the clerkship” and the percentage indicating the concept was “extremely important” or “moderately important”.

The majority of respondents identified lack of curricular time (81.9%) and faculty availability to teach (75.2%) as somewhat or major impediments to including clinical reasoning education in clerkships (Table 4). A minority of respondents identified perceptions that clinical reasoning cannot be taught (29.4%) or is too advanced (23.1%) as somewhat or major impediments.

**Table 4:** Degree to which specific barriers impede the inclusion of clinical reasoning activities in the clerkship phase of medical school.

Questionnaire item	Clerkship phase		
	Not an impediment n (%)	Somewhat of an impediment n (%)	A major impediment n (%)
Lack of faculty to teach CR^a^	71 (24.8)	124 (43.4)	91 (31.8)
Lack of curricular time	52 (18.2)	146 (51.1)	88 (30.8)
Perceptions that CR concepts are too advanced	220 (76.9)	59 (20.6)	7 (2.5)
Perceptions CR cannot be taught	202 (70.6)	78 (27.3)	6 (2.1)

^a^CR = clinical reasoning

## Discussion

This study broadens the lens on the current state of clinical reasoning education across clinical clerkship specialties. A prior study limited to a single clerkship discipline found that a structured curriculum in clinical reasoning was needed, and lack of curricular time and faculty expertise were the biggest barriers to teaching clinical reasoning [13]. We found that these needs extend throughout multiple clerkship specialties.

Improving education in clinical reasoning has been proposed as a mechanism for improving diagnosis and reducing errors [1, 17], but there remains a need to expand curricula to address gaps between important concepts and how many clerkships teach these concepts. Most respondents were familiar with most of the clinical reasoning concepts in our survey. The least familiar concepts were the more general conceptual frameworks (dual process theory and Bayesian reasoning). These frameworks, as well as the use and limitations of heuristics, accounted for the largest gaps between importance and coverage in clerkships. While we did not ask about other emerging conceptual frameworks or theories, e.g. situated cognition, distributed cognition, and embodied cognition, our findings suggest that faculty development in foundational theories and a shared lexicon may be beneficial.

Many faculty may be familiar with clinical reasoning conceptual frameworks but may not be familiar with the terminology used to describe them. In an analysis of 79 clinical problem solving exercises in four clinical journals, the clinical reasoning terms most often used in the publications were discordant with the clinical reasoning terms prioritized by a group of medical educators [18]. In another review of 625 papers on clinical reasoning in health professions education, 110 different terms were found to describe clinical reasoning [19]. Developing a shared lexicon of clinical reasoning concepts would provide a foundational step in moving clinical reasoning education forward [20].

It is possible that respondents were familiar with some components of clinical reasoning conceptual frameworks, but not the frameworks themselves. For example, in a 2015 survey of internal medicine clerkship directors, estimation of pre-test probabilities was selected among the most important clinical reasoning topics for students to understand upon entering the clerkship [13]. Pre-test probability estimation is the first step in Bayesian reasoning, yet Bayesian reasoning was rated one of the least important or familiar concepts in our survey. Similarly, the use and limitations of heuristics is central to understanding the risk of error with type I reasoning in the dual process theoretical framework.

Faculty development is critical to elevating the teaching of important clinical reasoning concepts across the clerkships. For example, in our study problem representation and illness scripts were identified as the most important concepts to teach in clinical clerkships. In a faculty development study, a single case-based, interactive workshop followed by role-play exercises resulted in significant improvement in faculty’s use of problem representation and illness scripts when teaching [21].

Because clinical reasoning is content- and context-specific, it needs to be taught across all specialties, not just in an isolated course. We suggest integrating accessible, well-designed curricula aligned with students’ stage of training and which support independent learning to mitigate the barriers of curricular time and faculty expertise. Several educators have described specific tools, such as concept maps, contrasting cases, case conferences, virtual patients, and other curricular innovations to meet these goals at the pre-clerkship and clerkship level [22–30]. The need for coordinated curricula is not unique to US medical schools; a recent consensus statement from the United Kingdom argues for the need to implement an explicit longitudinal medical school clinical reasoning curriculum and reinforces the need for improving faculty development on teaching and assessing clinical reasoning, highlighting several of the clinical reasoning topics prioritized in our study [31].

There were several limitations to our study. The overall response rate was low and varied widely among specialties, so we cannot make comparisons across the specialties, but instead utilized the aggregate data. One specialty (psychiatry) had a moderate response rate, but modifications to the survey precluded inclusion of data in the aggregate analyses. Another specialty (surgery) did not participate in the study, and surgically oriented subspecialties overall are underrepresented. When examined separately, the results (S3 Appendix) relating to the importance of clinical reasoning concepts paralleled those reported in this study. It’s also not clear if non-respondents differed significantly from respondents. Finally, due to space limitations, we could not survey participants about all clinical reasoning concepts of interest, especially emerging theories such as situated cognition and distributed cognition [7–9].

Based on our results, we recommend the following: (1) develop a shared lexicon for describing important clinical reasoning concepts that is used in clinical, classroom, virtual teaching and publications; (2) develop both specialty-specific and cross-disciplinary curricula that are coordinated horizontally, across all required clinical clerkships, and vertically, supporting a developmental progression in clinical reasoning; and (3) collaborate with specialty-specific educator societies to create faculty development programs for all required clinical clerkship specialties, and include conceptual frameworks for clinical reasoning in these faculty development programs.

## Supporting information

S1 Appendix Survey tool. (DOCX)

S2 Appendix Importance of teaching clinical reasoning concepts during the clerkship and degree to which they are included in clerkship phases (non-clerkship leaders). (DOCX)

S3 Appendix Importance of clinical reasoning concepts during the clerkship for ADMSEP. (DOCX)

S1 Dataset (XLSX)
