# Correction: Clinical reasoning education in the clerkship years: A cross-disciplinary national needs assessment

**DOI:** 10.1371/journal.pone.0307054

**Published:** 2024-07-09

**Authors:** Jonathan G. Gold, Christopher L. Knight, Jennifer G. Christner, Christopher J. Mooney, David E. Manthey, Valerie J. Lang

The fourth author’s middle initial is incorrect. The correct name is: Christopher J. Mooney. The correct citation is: Gold JG, Knight CL, Christner JG, Mooney CJ, Manthey DE, Lang VJ (2022) Clinical reasoning education in the clerkship years: A cross-disciplinary national needs assessment. PLOS ONE 17(8): e0273250. https://doi.org/10.1371/journal.pone.0273250.
