# Supplementary material for: Clinical reasoning education in the clerkship years: A cross-disciplinary national needs assessment
Source: PLoS One. 2022 Aug 18;17(8):e0273250. doi: 10.1371/journal.pone.0273250 (PMC9387845; doi:10.1371/journal.pone.0273250)
Supplement: S1 Appendix — (DOCX) [file pone.0273250.s001.docx]

Appendix A: Survey Tool

Q1: How important is it to formally include teaching or learning activities that specifically address the following clinical reasoning concepts during the **CLERKSHIPS** portion of the medical school curriculum?

|  | **Not sure OR Unfamiliar with the concept** | **Not at all important** | **Moderately important** | **Extremely important** |
| --- | --- | --- | --- | --- |
| **Use of semantic qualifiers** (i.e. abstract, often binary terms that help narrow or specify the meaning of a symptom, sign, pathologic process or disease)  For example: acute vs. chronic, moderate vs. severe, or unilateral vs. bilateral |  |  |  |  |
| **Use of problem representations** (i.e. the clinician’s synthesis of key discriminating aspects of the history, exam, and data; often expressed as a summary statement)  For example: 60 yo year-old male man with history of type 2 diabetes and hypertension with acute onset of left-sided chest pain and diaphoresis |  |  |  |  |
| **Use of illness scripts** (i.e. physician’s mental representations of clinical findings associated with diseases)  For example: The representative findings for croup are: age 18 months to 3 years-old with barky cough, and stridor, and presentation from October to March) |  |  |  |  |
| **Dual-processing theory** (i.e. description of cognitive processes as the interplay of non-analytic and analytical reasoning approaches. or System 1 (fast, pattern recognition, experience-based) vs. System 2 (slow, deliberate, rational))  For example: An expert sees a swollen calf in a post-op patient and diagnoses a DVT (system 1). A novice learner looks at a swollen leg and needs to consider a variety of causes, associate with pathophysiology to consider DVT as a diagnosis (system 2). |  |  |  |  |
| **Use and limitations of heuristics** (i.e. mental short cuts or rules of thumb used subconsciously in approaching a problem)  For example: A physician immediately thinks of influenza in a patient presenting with fever during flu season |  |  |  |  |
| **Bayesian reasoning** (i.e. calculating the post-test probability as the product of the pre-test probability and the likelihood ratio)  For example: with a pretest probability of 25% for pulmonary embolus and a negative D-dimer test with a LR of 0.1, the post-test probability of PE is 3% |  |  |  |  |
| **Cognitive bias** (i.e. dispositions or preferences that can affect judgments and decisions in a subconscious manner.)  For example: narrowly focusing on a single feature (sore throat) to support the diagnostic hypothesis even if other or new information refute that diagnosis contradicts it (cough, lack of exudate, lack of fever) |  |  |  |  |

Q2: To what degree are each of the following clinical reasoning concepts included in formal teaching or learning activities in the **CLERKSHIPS** portion of the curriculum at your institution?

|  | **Not sure OR Unfamiliar with the concept** | **Not covered in the OVERALL CLERKSHIPS curriculum at all** | **Covered in the OVERALL CLERKSHIPS curriculum but not in my clerkship** | **Covered in my clerkship** |
| --- | --- | --- | --- | --- |
| **Use of semantic qualifiers** (i.e. abstract, often binary terms that help narrow or specify the meaning of a symptom, sign, pathologic process or disease)  For example: acute vs. chronic, moderate vs. severe, or unilateral vs. bilateral |  |  |  |  |
| **Use of problem representations** (i.e. the clinician’s synthesis of key discriminating aspects of the history, exam, and data; often expressed as a summary statement)  For example: 60 yo year-old male man with history of type 2 diabetes and hypertension with acute onset of left-sided chest pain and diaphoresis |  |  |  |  |
| **Use of illness scripts** (i.e. physician’s mental representations of clinical findings associated with diseases)  For example: The representative findings for croup are: age 18 months to 3 years-old with barky cough, and stridor, and presentation from October to March) |  |  |  |  |
| **Dual-processing theory** (i.e. description of cognitive processes as the interplay of non-analytic and analytical reasoning approaches. or System 1 (fast, pattern recognition, experience-based) vs. System 2 (slow, deliberate, rational))  For example: An expert sees a swollen calf in a post-op patient and diagnoses a DVT (system 1). A novice learner looks at a swollen leg and needs to consider a variety of causes, associate with pathophysiology to consider DVT as a diagnosis (system 2). |  |  |  |  |
| **Use and limitations of heuristics** (i.e. mental short cuts or rules of thumb used subconsciously in approaching a problem)  For example: A physician immediately thinks of influenza in a patient presenting with fever during flu season |  |  |  |  |
| **Bayesian reasoning** (i.e. calculating the post-test probability as the product of the pre-test probability and the likelihood ratio)  For example: with a pretest probability of 25% for pulmonary embolus and a negative D-dimer test with a LR of 0.1, the post-test probability of PE is 3% |  |  |  |  |
| **Cognitive bias** (i.e. dispositions or preferences that can affect judgments and decisions in a subconscious manner.)  For example: narrowly focusing on a single feature (sore throat) to support the diagnostic hypothesis even if other or new information refute that diagnosis contradicts it (cough, lack of exudate, lack of fever) |  |  |  |  |

Q3: What is your best estimation of the number of hours dedicated to formal clinical reasoning teaching or learning in the **CLERKSHIPS** portion of the curriculum. (don’t know, < 1 hour, 1-4 hours, > 4 hours)

Q4: Indicate the degree to which each of the following barriers impedes or would impede the inclusion of formal clinical reasoning teaching or learning activities in the **CLERKSHIPS** portion of the curriculum at your institution?

|  | **Not at all an impediment** to inclusion of clinical reasoning teaching or learning activities | **Somewhat of an impediment** to inclusion of clinical reasoning teaching or learning activities | **A major impediment** to inclusion of clinical reasoning teaching or learning activities |
| --- | --- | --- | --- |
| Lack of curricular time to add this content |  |  |  |
| Perceptions that clinical reasoning cannot be taught |  |  |  |
| Perception that these concepts are too advanced for clerkship students |  |  |  |
| Lack of faculty to teach this content |  |  |  |

Q5: What other barriers impede or would impede the inclusion of formal clinical reasoning teaching or learning activities in the **CLERKSHIPS** portion of the curriculum at your institution?

| **Comments:** *[free text field]* |
| --- |

Note: The next two questions refers to your familiarity with the clinical reasoning instruction occurring **BEFORE** students have started their clerkship curriculum.

Q6: How important is it to formally include teaching or learning activities that specifically address the following clinical reasoning concepts during the **PRE-CLERKSHIP** medical school curriculum?

|  | **Not sure OR Unfamiliar with the concept** | **Not at all important** | **Moderately important** | **Extremely important** |
| --- | --- | --- | --- | --- |
| **Use of semantic qualifiers** (i.e. abstract, often binary terms that help narrow or specify the meaning of a symptom, sign, pathologic process or disease)  For example: acute vs. chronic, moderate vs. severe, or unilateral vs. bilateral |  |  |  |  |
| **Use of problem representations** (i.e. the clinician’s synthesis of key discriminating aspects of the history, exam, and data; often expressed as a summary statement)  For example: 60 yo year-old male man with history of type 2 diabetes and hypertension with acute onset of left-sided chest pain and diaphoresis |  |  |  |  |
| **Use of illness scripts** (i.e. physician’s mental representations of clinical findings associated with diseases)  For example: The representative findings for croup are: age 18 months to 3 years-old with barky cough, and stridor, and presentation from October to March) |  |  |  |  |
| **Dual-processing theory** (i.e. description of cognitive processes as the interplay of non-analytic and analytical reasoning approaches. or System 1 (fast, pattern recognition, experience-based) vs. System 2 (slow, deliberate, rational))  For example: An expert sees a swollen calf in a post-op patient and diagnoses a DVT (system 1). A novice learner looks at a swollen leg and needs to consider a variety of causes, associate with pathophysiology to consider DVT as a diagnosis (system 2). |  |  |  |  |
| **Use and limitations of heuristics** (i.e. mental short cuts or rules of thumb used subconsciously in approaching a problem)  For example: A physician immediately thinks of influenza in a patient presenting with fever during flu season |  |  |  |  |
| **Bayesian reasoning** (i.e. calculating the post-test probability as the product of the pre-test probability and the likelihood ratio)  For example: with a pretest probability of 25% for pulmonary embolus and a negative D-dimer test with a LR of 0.1, the post-test probability of PE is 3% |  |  |  |  |
| **Cognitive bias** (i.e. dispositions or preferences that can affect judgments and decisions in a subconscious manner.)  For example: narrowly focusing on a single feature (sore throat) to support the diagnostic hypothesis even if other or new information refute that diagnosis contradicts it (cough, lack of exudate, lack of fever) |  |  |  |  |

Q7: What is your level of familiarity with the clinical reasoning instruction occurring in the **PRE-CLERKSHIP** curriculum at your institution?

| Not at all familiar | Slightly familiar | Moderately Familiar | Extremely Familiar |
| --- | --- | --- | --- |
|  |  |  |  |

Q8: Please include any additional comments you have about clinical reasoning instruction for medical students in the **PRE-CLERKSHIP** and/or **CLERKSHIP** curricula.

| **Comments:** *[free text field]* |
| --- |

**Demographic Questions**

Q9: What is your role (if any) in your clerkship curriculum? (eg clerkship director, assistant or associate clerkship director, site director,, subinternship director, other (textbox))

Q10: How long is your current clerkship (< 6 weeks, 6 weeks to 8 weeks, > 8 weeks, other [longitudinal clerkship, interdisciplinary clerkship])

*Survey initially adapted from Rencic, Trowbridge, Fagan, Szauter, and Durning. Clinical Reasoning Education at US Medical Schools: Results from a National Survey of Internal Medicine Clerkship Directors. J Gen Intern Med. 2017 Aug 24. doi: 10.1007/s11606-017-4159-y.*
