# Supplementary material for: Clinical reasoning education in the clerkship years: A cross-disciplinary national needs assessment
Source: PLoS One. 2022 Aug 18;17(8):e0273250. doi: 10.1371/journal.pone.0273250 (PMC9387845; doi:10.1371/journal.pone.0273250)
Supplement: S2 Appendix — (DOCX) [file pone.0273250.s002.docx]

**Appendix B. Importance of teaching clinical reasoning concepts during the clerkship and degree to which they are included in clerkship phases (non-clerkship leaders)**

| Questionnaire item | Importance during clerkship (n=65) | | | | Included in clerkship (n=62) | | | | Gap* |
| --- | --- | --- | --- | --- | --- | --- | --- | --- | --- |
|  | Not sure or unfamiliar  n (%) | Not at all important    n (%) | Moderately important    n (%) | Extremely important    n (%) | Not sure or unfamiliar    n (%) | Not covered    n (%) | Covered elsewhere    n (%) | Covered in clerkship  n (%) | (%) |
| Use and limitations of heuristics | 2. (3.1) | 2 (3.1) | 32 (49.2) | 29 (44.6) | 25 (40.3) | 7 (11.3) | 12 (19.4) | 18 (29.0) | 64.8 |
| Dual-processing theory | 4 (6.2) | 8 (12.3) | 29 (44.6) | 24 (36.9) | 25 (40.3) | 13 (20.9) | 10 (16.3) | 14 (22.6) | 58.9 |
| Bayesian reasoning | 4 (6.2) | 8 (12.3) | 34 (52.3) | 19 (29.2) | 24 (29.3) | 10 (16.4) | 10 (16.4) | 17 (27.9) | 53.6 |
| Cognitive bias | 1 (1.5) | 3 (4.6) | 19 (29.2) | 42 (64.6) | 18 (29.0) | 9 (14.5) | 10 (16.1) | 25 (40.3) | 53.5 |
| Semantic qualifiers | 2 (3.1) | 1 (1.5) | 23 (35.3) | 39 (60.0) | 15 (24.2) | 8 (12.69) | 8 (12.9) | 31 (50.0) | 45.3 |
| Illness scripts | 1 (1.5) | 1 (1.5) | 22 (33.9) | 41 (63.1) | 12 (19.4) | 7 (11.3) | 8 (12.9) | 35 (56.5) | 40.5 |
| Problem representations | 2 (3.1) | 0 (0.0) | 9 (13.9) | 54 (83.1) | 14 (22.6) | 3 (4.8) | 3 (4.8) | 42 (67.7) | 29.3 |

* Gap refers to the difference between the percentage of respondents indicating that a concept was “covered in the clerkship” and the percentage indicating the concept was “extremely important” or “moderately important”.
