# Supplementary material for: Clinical reasoning education in the clerkship years: A cross-disciplinary national needs assessment
Source: PLoS One. 2022 Aug 18;17(8):e0273250. doi: 10.1371/journal.pone.0273250 (PMC9387845; doi:10.1371/journal.pone.0273250)
Supplement: S3 Appendix — (DOCX) [file pone.0273250.s003.docx]

**Appendix C.** I**mportance of clinical reasoning concepts during the clerkship for ADMSEP**

| Questionnaire item |  | | Importance during clerkship (n=77) | | | |
| --- | --- | --- | --- | --- | --- | --- |
|  | Not sure or unfamiliar  n (%) | Not at all important    n (%) | | Slightly important    n (%) | Moderately important    n (%) | Extremely important    n (%) |
| Semantic qualifiers | 9 (11.6) | 2 (2.6) | | 12 (15.6) | 17 (22.1) | 37 (48.5) |
| Problem representations | 4 (5.2) | 1 (1.3) | | 4 (5.2) | 17 (22.1) | 51 (66.2) |
| Illness scripts | 4 (5.2) | 0 (0.0) | | 5 (6.5) | 17 (22.1) | 51 (66.3) |
| Dual-processing theory | 10 (12.9) | 2 (2.6) | | 11 (14.3) | 29 (37.7) | 25 (32.5) |
| Use and limitations of heuristics | 6 (7.8) | 1 (1.3) | | 11 (14.3) | 29 (37.7) | 30 (39.0) |
| Bayesian reasoning | 8 (10.4) | 3 (3.9) | | 13 (16.8) | 30 (39.0) | 23 (29.9) |
| Cognitive bias | 5 (6.5) | 0 (0.0) | | 9 (11.7) | 22 (28.6) | 41 (52.3) |
|  |  |  |  |  |  |  |
